# Algerian Energy Policy: Perspectives, Barriers, and Missed Opportunities

**DOI:** 10.1002/gch2.201700134

**Published:** 2018-07-03

**Authors:** Saliha Haddoum, Hocine Bennour, Toudert Ahmed Zaïd

**Affiliations:** ^1^ Ecole Nationale Polytechnique Département de Génie Chimique Laboratoire de Valorisation des Energies Fossiles 10 Avenue Hassen Badi BP182 El‐Harrach 16200 Algiers Algeria

**Keywords:** barriers, energy policy, energy–water–food nexus, RE Program

## Abstract

Despite the uncertainties of the energy market impacted by the collapse of oil prices and a sharp increase in domestic energy consumption, Algeria continues to make huge efforts to bring energy access to its people. At the same time, the country is also witnessing a very slow start of the energy transition, which brings into question the energy‐intensive development model accused of contributing both to global warming and to the depletion of fossil resources in the medium and long term. Although quantified targets are set, the National Program for the Development of Renewable Energies (NPDRE) struggles to take off and is lagging behind. There is no doubt that the recent fall in oil prices will further complicate the achievement of this transition; still one may raise the question of whether other barriers to the NPDRE should not be removed. Admittedly, the Algerian State has set up an incentive mechanism based on feed‐in‐tariffs to boost the NPDRE, but this failed to attract potential investors. This paper seeks to analyze the reasons for these failures as well as other issues linked with the energy–water–food trilemma. There are plenty, but the heavily subsidized energy products appear to be the most disabling.

## 1 Introduction

The world of energy is facing unprecedented uncertainty. The global economic crisis of 2008–2009 has plunged energy markets around the world into turmoil and the pace at which the global economy is recovering will impact the energy outlook for the future. But it will be governments, and how they address the dual challenge of climate change and energy security, that will shape the future of energy in the longer term.^1^

Despite the uncertainties of the energy market and the falling oil prices, Algeria continues to make huge efforts to bring energy access to its people. Electricity needs are rising very rapidly, with an average increase of 6% over the ten last years.

Lower oil prices are already having a large impact on the deployment of renewable energy technologies in the power sector, even if policymakers keep on providing the necessary market rules, policies, and subsidies. Indeed, the integration of renewable energies into the national energy mix is a major challenge for the preservation of fossil fuels, the diversification of electricity production chains, and the contribution to sustainable development. Algeria is also committed to reduce its greenhouse gas (GHG) emissions by 2020–2030. An incentive mechanism based on feed‐in tariffs is established by regulation since 2014.^2^ A Renewable Energy Fund was also planned to be financed through a 1% levy on oil revenues. Despite all these efforts, the RE Program is slow to materialize. This paper examines some of the barriers that explain this shortcoming and advocates a better energy demand management and pricing policy.

## 2 The Renewable Energy Program

Algeria's government is committed to shift away from fossil fuels to renewable energy sources both to preserve the declining fossil resources and because this is the best long‐term solution to achieve environmental objectives. This aims also to move away from the high dependence on natural gas by increasing the share of electricity generated by renewables and cogeneration.

Indeed, the three countries of the North African Maghreb region, Morocco, Algeria, and Tunisia, are showing increased efforts to integrate renewable electricity into their power markets.^3^ **Table** **1** below shows recently announced MENA countries RE targets. How fast these targets will be met depends on the commercial viability of the different renewable energy technologies and their competitiveness compared with fossil fuels. Cost effectiveness remains a decisive factor in encouraging developing countries to use clean generation technologies. Other factors are directly linked with the energy policy and practices in place. Countries that have seen the greatest successes usually have a high level of government intervention in terms of market alterations and diversions of community and industry away from systems that rely on fossil fuels.^4^

**Table 1:** MENA countries renewable energy targets^4^

Country	Targets	Type of technology
Algeria	15% 2020	37% solar (PV and CSP)
	40% 2040	3% wind
Libya	7% 2020	Solar PV
	10% 2025	
Morocco	42% 2020	Solar PV and CSP
Tunisia	11% 2016	Wind 1.7 GW 2030
	25% 2030	Solar PV 1.5 GW 2030
	40% installed 2030	Solar CSP 500 MW
Egypt	20% 2020	8% Solar PV & CSP 12% Wind
Saudi	50% 2032	Total 54 GW
Arabia		Solar PV & CSP 42 GW Wind 9 GW
UAE	Dubai 5% 2030	Solar PV
	Abu Dhabi 7% 2020	Solar CSP
	20% 2030 (2.5 GW)	Wind

Algeria has been too slow in implementing its renewable energy program. This is a consequence of its strong reliance on fossil energies to support the economy along with heavily subsidized energy products. In 2015, the authorities even lowered the objectives of the former NPDREP, which aimed to achieve 40% of Renewables in the energy mix by 2040. 27% RE in total electricity production are thus planned by 2030. The targets per technology are set according to two phases as outlined in **Table** **2**.

**Table 2:** Detailed Algerian RE targets^5^

Source	First phase 2015–2020	Second phase 2021–2030	Total [MW]
Solar PV	3000	10 575	13 575
Wind	1010	4000	5010
CSP	–	2000	2000
Cogeneration	150	250	400
Biomass	360	640	1000
Geothermal	5	10	15
Total	4525	17 475	22 000

So far, achievements are rather modest compared to the forecasts:

‐ A solar power plant (1.1 MW) and a wind farm (10 MW) commissioned in 2014 in Ghardaïa and Adrar respectively, in addition to the Hassi R'mel hybrid plant (150 MW) of which 25 MW come from photovoltaics;

‐ A new wind farm of 20 MW and a new solar power plant (3 MWe) were added to these achievements at Adrar in 2015 and 2016, respectively. Thus, to date, roughly 60 MW are produced (PV, Wind). A significant step was made in 2015 when the country revised its FIT program to provide additional support for the deployment of wind and solar PV projects as well as Combined Heat and Power generation. This later is intended to make natural gas savings. But despite all these supporting policies, the NPDRE is behind schedule. Recently, Algeria launched a tender for the production of 400 MW of electricity from solar PV in order to bridge the gap between RE Program forecasts and the actual achievements.

Algeria recently reiterated its commitment to reduce its GHG emissions by 7% by 2020–2030 under the Paris Climate Agreement adopted in 2015, which aims to maintain the rise in temperature average of the Earth below 2 °C. This GHG reduction could reach 22% if Algeria receives the necessary international support, by 2030. So far, renewable electricity generation has not reached a level that allows a significant contribution to energy‐based carbon dioxide emissions reduction target.^6^

## 3 Brief Diagnosis

A brief diagnosis of the Algerian energy situation can shed some light on the reasons of the shortcomings and delays in the Renewable Energy program deployment. Algeria is currently in a context of accelerating its energy consumption, together with a hydrocarbon production, which has seen successive declines from 2007 and a slight recovery in the last two years. Indeed, national energy consumption has more than doubled since 2000, rising from 28 to 58 Mtoe in 2015. This represents an average growth of 5% per year, with acceleration over the last five years at 6%. Over the same period 2000–2015, the gross domestic product at constant prices increased by more than 70%, from 4123 to 7029 GDA, which represents an average annual growth rate of 3.6%. This obviously indicates deterioration in the energy efficiency of the economy. The national energy intensity, expressed as the amount of energy needed to produce a unit of GDP, has evolved over the last five years at an accelerated pace of 2.4% per year, compared with 0.8% over the entire period 2000–2015.

This growth in consumption combined with the fall in production has led to a drop in energy exports in recent years (nearly 31% since 2005).

Under such conditions, where consumption is mainly driven by a nonproductive sector, that of residential, energy efficiency is likely to play an important role and could result in considerable savings if sustained energy efficiency measures are implemented.

On the other hand, the energy sector has made considerable efforts and investments in the development and improvement of access to energy throughout the country.

For electricity, production capacity has increased substantially, to almost triple from 5.9 GW in 2000 to more than 17.2 GW in 2015. Production has recorded an average annual change of 6.5%, to reach about 65 TWh in 2015 compared to 25 TWh in 2000. In the same time, one should point out that energy prices in all its forms have not been revised since 2006, despite rising costs on the one hand and rising household incomes, on the other hand, which does not encourage the development of activities and/or reduce the scope of energy efficiency measures.

Indeed, national performances in terms of economic energy efficiency leave much to desire, despite the energy efficiency programs adopted by the public authorities since 1999.

On average, oil consumption has increased by 5% per year over the last decade. Although rising incomes account for most of this upward trend in domestic consumption, low energy prices have also contributed significantly to higher levels of energy consumption. The price of heavily subsidized energy has been a concern for several years now.

## 4 Barriers to Energy Transition and RE Deployment

It is a fact that the energy sector is the backbone of the Algerian economy. Therefore, energy planning requires a thorough analysis of the links between this sector and the rest of the economy. In regards to national objectives, areas for further analysis should include the links between food security, water security, and agriculture and the impact of policies regarding prices, taxes on the economy. The lack of intersectoral coordination largely explains the shortcomings in many sectors of the national economy. The lack of data and the laxity engendered by the heavy reliance on hydrocarbons are also to be considered. Barriers to renewable energy deployment obviously include subsidies for conventional forms of energy, lack of skills or information, poor market acceptance as well as structural and institutional factors.

### 4.1 Heavily Subsidized Energy Products

Energy policy reflects the way the government addresses energy development issues, including energy production, distribution, and consumption. The attributes of an energy policy generally include legislation, international treaties, pricing, incentives, taxation, and energy conservation guidelines. Pricing is the most effective tool of energy demand management, especially in the medium and long run.^7^

Until 2016, practical policies have focused on increasing subsidies for renewable energy rather than reducing subsidies for fossil fuels. In fact, the 2016 budget even included an increase in the cost of direct subsidies, accounting for 23% of total public spending. Faced with the shrinking oil revenues, this trend seems henceforth financially unsustainable. Judicious reforms could have been undertaken during the period from 2004 to 2014 which saw the oil prices spike from $35 to $130/barrel. Instead, they have led to a massive rise in salaries. This is a clear case of missed opportunities that marked so much the last decade. Successive governments have avoided addressing the problems at source for political reasons. They were more inclined to simply manage oil incomes to ensure social peace rather than tackling the challenge of sustainable development.

Subsidies are certainly useful for stimulating specific economic sectors or helping segments of the population, reducing poverty and increasing access to energy, but unfortunately they have also many negative effects and encourage waste. A 2016 study estimated that global fossil fuel subsidies were $5.3 trillion in 2015, which represents 6.5% of global GDP,^8^ although such figures are very difficult to estimate. In 2014, Algeria subsidies were estimated at $20 billion.^9^

The fall in oil revenues and the grim outlook for the national economy have finally triggered a raft of reforms intended to lower subsidies. Diesel and gasoline prices were raised 48% and 54%, respectively, since 2016. Electricity tariffs have also been raised but this does not seem to curb the soaring consumption trend. Indeed, despite these increases in fuel and electricity tariffs, they remain among the lowest in the world. With such low tariffs, all the measures taken to maintain grid utilization within the limits of capacity constraints are proving difficult. The electricity company (Sonelgaz) carried out large information campaigns to encourage users to consume electricity rationally. Incentives are given through a system of dynamic pricing and in particular through tariffs that are significantly higher during periods of high network usage. But this is not the only issue faced by the Company which strives to recover receivables from its customers among the bad payers. Unpaid invoices amounting maybe to more than 1000 GDA seriously endangers the Company's perenniality. The fact that the highest proportion of these receivables is held by the government and the public sector sends obviously a bad signal. This distortion also explains the widely held belief that many of the public services should be free.

### 4.2 Lack of Foresight and Overall Policy Coherence

Algeria like some other countries in the region continue to adhere to particularly narrow energy policies, pursuing a solution to one type of challenge that often occurs at the expense of solutions to other challenges. Energy pricing policy should not focus on a particular issue but should consider trade‐offs. All policy packages involve trade‐offs. Without a coordinated strategy that treats the challenges relating to electricity, transportation, agriculture, waste and water, and climate change as connected, unanticipated consequences can result. Expeditious reforms and decisions have often led to catastrophic results.

Undeniably, this lack of foresight stems from the reliance on fossil energy revenues since decades. The missed opportunities to which we were referring are not merely a vision but something that has hampered a sustainable development trajectory. The era of high oil incomes is well behind us and in any case the soaring domestic energy consumption will sooner or later leave no room for export. Algeria's export potential could double by 2030 and remain above 60 billion m^3^ by 2040.^10^ But much gloomy prospects concerning gas production project a reduction in Sonatrach gas exports to some 15 billion m^3^ per year by 2030 in a scenario of moderate growth in domestic demand, or even disappear in a higher demand growth scenario.^11^

Faced with this decline in oil incomes, the country's energy security is at stake. Other strategic sectors will be impacted too, since energy is inherently a cross‐cutting issue, affecting all aspects of economic and social life. Agriculture is probably one of the most crucial sectors related to energy and it holds the key to the food security challenge that the country is seeking to meet. In addition, agriculture is strongly dependent on water resources. Algeria, like many countries of the Mediterranean region, faces a major water crisis that may be exacerbated with global warming and it relates directly to food and energy security.

The future development of water resources will require high energy consumptions, for seawater desalination, wastewater treatment, and the introduction of drip irrigation. Thus, more coordinated planning and action will be needed between the water and energy sectors to prevent a further worsening of the water deficit.^12^ Integrated thinking on energy and water is indeed essential to mitigate future stresses.

### 4.3 Regional Security Issues

Heavily subsidizing energy products create incentives for smuggling to neighboring countries. Smuggling, on the other hand, finances illegal activities and in some cases terrorism and rebellion.^13^ Regional insecurity have displaced thousands both internally and into neighboring countries. Terrorist activities occurring in Libya and Mali continue to displace individuals, placing strain on Algerian border security. Various separate terrorist attacks on oil installations plotted by militants seeking to enter the country from Libya have already been foiled.^14^

### 4.4 Energy, Water, and Food Wastes and Losses

Waste of resources is a major problem that must be addressed urgently. Water and food are wasted because of inefficient policies and they are closely linked to highly subsidized energy products. Simply stating that the water–energy–food nexus is unsustainable is underestimating the issue.^15^ Algeria is under heavy water stress, yet water is subsidized. Subsidized electricity is used to produce desalinated water, which is in turn subsidized. Whenever food is wasted, all the natural resources used for cultivation, processing, packaging, and transportation are also wasted. For example, when an apple is wasted, 70 L of water used for its production are also wasted.^16^ It is clear, therefore, that subsidies have many drawbacks and constitute a major barrier to sustainable development trajectories.

## 5 Conclusion and Policy Implications

Energy subsidies are intimately intertwined with the country's economy, which makes any reform extremely complex. Algeria's dual challenge is to divert the country's economy away from oil and gas, while at the same time amortizing the social impact of energy reforms.

Algeria's fuel subsidy affects the country's economy in two ways: in addition to reducing export revenues, it represents a reckless expense at a time when the country is facing record fiscal deficits following the drop in oil prices.

The economic growth objective requires that pricing policy should promote economically efficient allocation of resources, both within the energy sector and between it and the rest of the economy.^7^ Efforts targeting just one level of response are often useless and will allow crucial barriers to persist.^17^

Energy pricing policy, particularly energy taxation, needs to be complemented by other instruments of energy policy, which are specific to consumer segments or technologies to target barriers to the implementation of the efficiency potential.

One can argue that energy prices which send the correct signals about the true costs of energy to consumers are a necessary condition to achieve the targets, but they are not sufficient, since there are a large number of barriers and market imperfections which hinder the realization of the cost effective potentials particularly for energy efficiency but also for renewable energies. Nonetheless, administration should identify and educate the public about energy policies that make wise tradeoffs across issues. On the other hand, the changes brought by any reform would also require a credible commitment from the government to ensure that the associated costs and benefits will be fairly shared.^18^

It appears clearly that there is an urgent need for a transition to a new energy model in Algeria which, in addition to investing in renewable energies, must urgently remedy the soaring domestic consumption of energy products, and a subsidy policy that is both costly and harmful. There is also an urgent need to ensure greater transparency in the management of public resources that are often misused and diverted, including subsidized ones.

There is a need for a more open and interdisciplinary dialogue on the nexus of food security, water security, and energy security. The water security concerns link directly with the achievement of food and energy security but more importantly, also to broader regional security and peace.^15^

## Conflict of Interest

The authors declare no conflict of interest.
